# Paratubal Leiomyoma Mimicking Ovarian Malignancy: A Case Report and Literature Review

**DOI:** 10.3390/diagnostics16020218

**Published:** 2026-01-09

**Authors:** Wen-Lin Hsieh, Dah-Ching Ding

**Affiliations:** 1 Department of Obstetrics and Gynecology, Hualien Tzu Chi Hospital, Buddhist Tzu Chi Medical Foundation, Tzu Chi University, Hualien 970, Taiwan; 108311125@gms.tcu.edu.tw; 2 Institute of Medical Sciences, Tzu Chi University, Hualien 970, Taiwan

**Keywords:** fallopian tube, leiomyoma, paratubal, malignancy, adnexa

## Abstract

**Background and Clinical Significance**: A paratubal leiomyoma is an exceptionally rare benign smooth muscle tumor arising from paratubal tissue, with only sporadic cases reported in the literature. **Case Presentation**: We present the case of a 72-year-old postmenopausal woman with intermittent spotting for three months. A pelvic examination revealed a retained intrauterine device, which was removed along with an old sanguineous discharge. A transvaginal ultrasound demonstrated a complex left adnexal mass with calcifications, and computed tomography (CT) confirmed a 7.8 × 5.5 × 4.7 cm lesion suggestive of an ovarian malignancy. Tumor markers showed mildly elevated CA-125 and carcinoembryonic antigen (CEA) levels. Endometrial sampling using a hysteroscopy and curettage revealed hyperplasia without atypia. The patient underwent a total laparoscopic hysterectomy with a bilateral salpingo-oophorectomy. A diagnostic laparoscopy revealed a well-circumscribed solid mass arising from the mesosalpinx, separate from the ovary and fallopian tube and consistent with a paratubal mass, which was successfully excised laparoscopically. Frozen sections suggested a fibroma, and the final pathology confirmed a paratubal leiomyoma with hyalinization, accompanied by adenomyosis and simple endometrial hyperplasia. The patient recovered uneventfully, and the 6-month follow-up showed no recurrence. This case highlights the diagnostic challenge of differentiating paratubal leiomyomas from ovarian tumors based on imaging alone. Histopathological examination is essential for confirmation. **Conclusions**: Awareness of paratubal leiomyomas as a differential diagnosis may prevent overtreatment and guide the appropriate surgical management of postmenopausal women presenting with adnexal masses.

## 1. Introduction

A paratubal leiomyoma is an exceptionally rare benign smooth muscle tumor arising from the paratubal tissue. The literature on this condition is limited to case reports and small case series, with no population-based studies or epidemiological data quantifying their prevalence [1,2]. Most of the available data have focused on parasitic leiomyomas or paratubal cysts, rather than paratubal leiomyomas. Paratubal cysts constitute approximately 7–10% of adnexal masses in pediatric and adolescent populations; however, this prevalence does not apply to paratubal leiomyomas, which are distinct entities and are much less common [3,4]. Consequently, the prevalence of paratubal leiomyomas has not yet been established in the medical literature.

Paratubal leiomyomas are most often diagnosed intraoperatively or by histopathological examination after the surgical excision of an adnexal mass. Imaging modalities, such as transvaginal or transabdominal ultrasound, may reveal a solid, well-circumscribed adnexal mass separated from the ovary and uterus; however, preoperative differentiation from other adnexal masses (e.g., ovarian fibromas and paraovarian cysts) is challenging because of overlapping sonographic features and the rarity of the entity [5].

The current guidelines for evaluating adnexal masses emphasize that serum CA-125 may aid in risk stratification, but has a limited specificity, particularly in premenopausal women, and should be interpreted in conjunction with imaging findings and the clinical context [6,7].

A definitive diagnosis requires the histopathological confirmation of a leiomyoma composed of interlacing bundles of smooth muscle cells without atypia, necrosis, or significant mitotic activity, with clear anatomic separation from the uterus and ovary [8].

We report the case of a 72-year-old woman with a paratubal leiomyoma mimicking an ovarian malignancy who underwent a laparoscopic total hysterectomy and a bilateral salpingo-oophorectomy (LTH + BSO).

## 2. Case

A 72-year-old postmenopausal woman presented with intermittent spotting that had persisted for three months.

A pelvic examination revealed an old intrauterine device (IUD) string, and the removal of the retained IUD resulted in the release of an old sanguineous discharge.

Initial transvaginal ultrasonography revealed a complex solid mass with focal calcifications in the left adnexal region, suggesting a teratoma or ovarian malignancy. The uterus appeared enlarged, and the endometrium was markedly thickened. Subsequent computed tomography confirmed a left adnexal mass measuring 7.8 × 5.5 × 4.7 cm.

Tumor markers showed mildly elevated levels of CA-125 (35.4 U/mL; normal value: 35) and carcinoembryonic antigen (CEA) (4.1 U/mL; normal value: 3.5), with normal levels of squamous cell carcinoma antigen (SCC) and CA19-9.

A Pap smear revealed ASC-US (atypical squamous cells of undetermined significance), and high-risk HPV (human papillomavirus) testing was negative.

After progesterone withdrawal, transvaginal ultrasonography revealed persistent endometrial thickening with intrauterine fluid.

A hysteroscopy with dilation and curettage was performed, and a pathological examination demonstrated endometrial hyperplasia without atypia. The patient was then admitted for further evaluation and management.

The patient’s past medical history was significant only for hypertension under regular treatment. She had experienced menopause at 45 years of age. The obstetric history included two term spontaneous vaginal deliveries.

**Figure 1:**
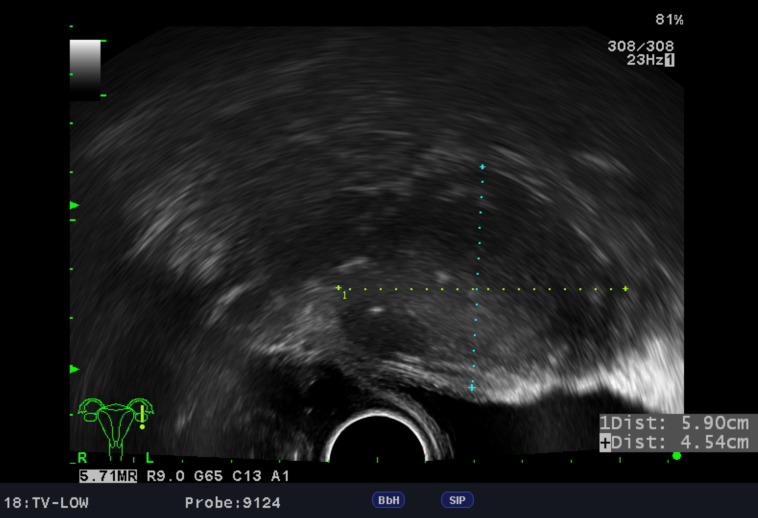
Transvaginal ultrasound of the left adnexal tumor (sagittal view). 1: Longitudinal diameter, +: Transverse diameter.

**Figure 2:**
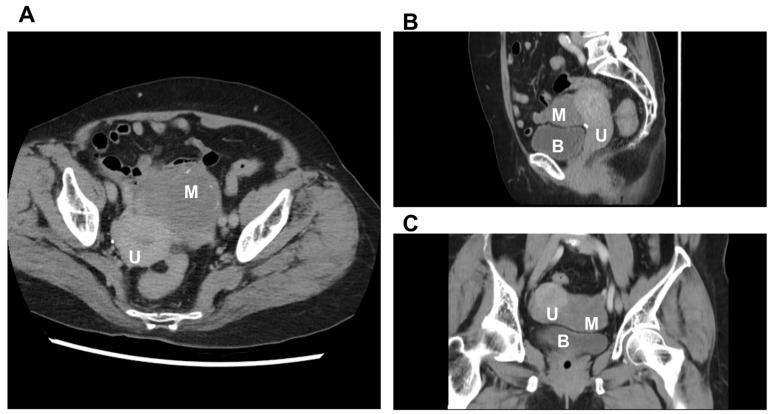
Computer tomography of the left adnexal tumor. (**A**) Axial view, (**B**) sagittal view, and (**C**) coronal view. U: uterus, M: leiomyoma, and B: bladder.

Imaging supported the initial suspicion of an adnexal tumor. Ultrasonography revealed a complex mass with calcifications (Figure 1), while computed tomography (CT) confirmed a 7.8 cm solid hypovascular tumor with calcified flecks in the left adnexal region and the rightward displacement of the uterus (Figure 2). Follow-up endometrial evaluations, including a hysteroscopy and curettage, revealed endometrial hyperplasia without atypia.

**Figure 3:**
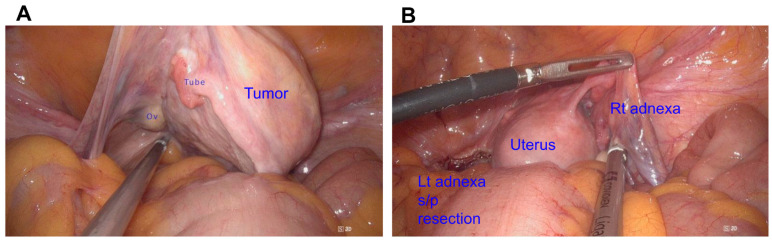
Laparoscopic view of surgical fields. (**A**) Before resection of left adnexa. (**B**) After resection of left adnexa: Ov: ovary. Tube: fallopian tube. Tumor: paratubal leiomyoma. Lt: left, Rt: right. s/p: status post.

The patient subsequently underwent a laparoscopic total hysterectomy with a bilateral salpingo-oophorectomy (LTH + BSO). Laparoscopic exploration revealed a well-circumscribed, solid mass located in the left paratubal region, distinct from the uterus, ovaries, and fallopian tubes (Figure 3A). The uterus demonstrated a normal contour without evidence of fundal or subserosal myomas (Figure 3B). Both the fallopian tubes and ovaries appeared grossly normal. The mass was connected by a narrow pedicle to the paratubal soft tissue and showed no direct attachment to the uterine serosa or tubal wall. The complete excision of the lesion was achieved without breaching the uterine or tubal structures (Figure 3B).

The estimated intraoperative blood loss was minimal, and the patient’s postoperative course was uneventful. A frozen-section analysis of the left ovary suggested a fibroma, and the final pathology was SMA (+), calretinin (−), and inhibin (−), confirming a paratubal leiomyoma with hyalinization accompanied by adenomyosis and simple endometrial hyperplasia. The patient was discharged in stable condition, with arrangements for outpatient follow-up.

Microscopically, the resected mass consisted of benign spindle cells with extensive hyalinization. Immunohistochemical staining was positive for smooth muscle actin and negative for calretinin and inhibin, which supported the diagnosis of leiomyoma with hyalinization. Masson’s trichrome staining further highlighted the presence of hyalinized regions.

At the 6-month follow-up visit, the vaginal stump appeared clean, although a small amount of white discharge was noted. A transvaginal ultrasound demonstrated a postoperative anatomy consistent with a hysterectomy and BSO, with no uterus or ovaries and no evidence of pelvic ascites.

## 3. Discussion

### 3.1. Previous Case Report

A literature review revealed that fallopian tube leiomyomas are exceedingly rare, with the published reports generally limited to isolated case descriptions. Fallopian tube leiomyomas may arise in any segment of the fallopian tube, most commonly in the isthmus [8]. They are typically solitary and small at the time of diagnosis [8]. Owing to the absence of characteristic early symptoms or signs, fallopian tube leiomyomas are frequently overlooked or incidentally detected during imaging or surgery [8]. Fallopian tube leiomyomas originate from the smooth muscle layer of the tubal wall, whereas paratubal leiomyomas arise from adjacent mesenchymal or smooth muscle tissues of the paratubal structures, such as the mesosalpinx, without the direct involvement of the fallopian tube itself. Previous reports have mostly described fallopian tube leiomyomas. Yang et al. (2007) documented a primary tubal leiomyoma with characteristic ultrasound findings [9]. Li et al. (2018) highlighted MED12 mutations in adnexal leiomyomas, suggesting a molecular basis similar to that of their uterine counterparts [10]. Additional reports include those by Misao et al. (2000) [11] and Joshi et al. (2019) [12], with the latter notable for coexistence with an ectopic pregnancy. Sharma et al. (2016) described a large cellular leiomyoma in a broad ligament that mimicked an ovarian tumor [13]. Sun et al. (2020) reported a lipoleiomyoma with degenerative changes [14]. Recently, Wu et al. (2024) presented a massive cystic variant that caused significant abdominal distension [8]. Compared with these previous reports, our case involved an older postmenopausal woman with a solid paratubal mass mimicking an ovarian malignancy, which was histologically confirmed as a leiomyoma with hyalinization without the involvement of the fallopian tube smooth muscle. This summary highlights the variable presentations, imaging features, and pathologies of tubal leiomyomas, underscoring the diagnostic challenges and the importance of histopathological confirmation (Table 1).

### 3.2. Differential Diagnosis of Fallopian Pathology

#### 3.2.1. Benign Tumors of the Fallopian Tube

Benign tumors of the fallopian tube constitute the least frequently encountered group of tubal neoplasms [15]. Their true incidence remains difficult to determine because of the scarcity of documented cases and the frequent misclassification of adnexal lesions originating from the ovaries [1,2]. Paratubal or paraovarian leiomyomas are extremely rare and are believed to arise from smooth muscle cells within the paratubal tissue or mesosalpinx [16]. Other smooth muscle tumors, including tubal leiomyomas and angioleiomyomas, have been reported, but they remain anecdotal in the literature [17]. Stromal and mesenchymal lesions such as fibromas, thecomas, and lipoleiomyomas are similar, but may appear as solid adnexal masses during ultrasound evaluation, potentially triggering concern for an ovarian fibroma or malignancy [18]. Benign epithelial tumors such as serous or mucinous cystadenomas, papillomas, or adenofibromas of tubal origin have also been described, but represent a very small proportion of adnexal masses [19]. Tumor-like lesions, including Walthard cell nests [20], endosalpingiosis [21], or paratubal cysts, often mimic neoplasms radiologically or macroscopically [22]. However, they are usually asymptomatic and are frequently identified incidentally during surgery or a histopathological examination. Hydatid cysts of Morgagni, one of the most common paratubal cystic lesions, may cause adnexal fullness or torsion, but rarely exhibit solid components [23]. The diagnostic challenge lies in differentiating these benign tumors from ovarian lesions preoperatively, because transvaginal ultrasonography often lacks the resolution necessary to reliably determine the precise origin of an adnexal mass, and the fallopian tube is frequently difficult to visualize in postmenopausal patients [5]. In clinical practice, benign tubal tumors are typically discovered intraoperatively when an adnexal mass is removed under suspicion of ovarian pathology [24]. Accordingly, a definitive diagnosis depends on histopathology demonstrating smooth muscle bundles, stromal composition, or epithelial features without atypia or malignant transformation [25]. Awareness of benign tubal tumors is important for making a clinical differential diagnosis to avoid excessive surgical interventions, especially in older women or those seeking fertility preservation.

In our case, immunohistochemistry (IHC) showed SMA (+), calretinin (−), and inhibin (−), which were compatible with leiomyomas. SMA positivity supports a smooth muscle origin, which is characteristic of leiomyomas [26]. In contrast, calretinin and inhibin are commonly expressed in sex cord stromal and mesothelial tumors; thus, their absence helps exclude ovarian stromal neoplasms, such as thecomas or fibromas, as well as mesothelial lesions [27]. Immunohistochemistry is valuable for distinguishing paratubal leiomyomas from other adnexal tumors, with a profile of SMA positivity, calretinin negativity, and inhibin negativity supporting a smooth muscle origin and confirming a leiomyoma.

#### 3.2.2. Primary Malignant Tumors of the Fallopian Tube

Primary malignant tumors of the fallopian tube are rare, accounting for less than 1% of gynecologic malignancies [24]. However, their clinical relevance has increased owing to emerging evidence linking high-grade serous carcinoma (HGSC) to the distal fallopian tube’s fimbrial epithelium [24]. HGSC is the most common primary tubal malignancy, whereas low-grade serous carcinoma, endometrioid carcinoma, mucinous carcinoma, clear cell carcinoma, and serous borderline tumors have been documented, but are significantly less frequent [28]. Non-epithelial malignancies, such as leiomyosarcoma, endometrial stromal sarcoma, or malignant mixed Müllerian tumors, are exceedingly rare and pose significant diagnostic difficulties because their presentation overlaps with ovarian or uterine malignancies [29]. Clinically, fallopian tube carcinomas often present with nonspecific symptoms such as pelvic pain, abnormal uterine bleeding, vaginal discharge, or an adnexal mass similar to ovarian cancer [30]. The classic triad of fallopian tube carcinomas—intermittent serosanguineous discharge, pelvic pain, and an adnexal mass—is infrequently observed in practice [31]. Imaging findings using ultrasonography or computed tomography typically reveal a complex adnexal mass, solid components, or papillary projections. However, these characteristics are not sufficiently specific to distinguish primary tubal cancer from ovarian neoplasms [32]. Magnetic resonance imaging (MRI) may offer better tissue characterization; however, even advanced imaging modalities may not reliably identify the tubal origin preoperatively [33]. Tumor markers such as CA-125 are often elevated, particularly in HGSC, but remain nonspecific [34]. Therefore, a definitive diagnosis relies on postoperative pathological criteria, including the demonstration of intraepithelial carcinoma within the tubal epithelium and a dominant tumor focus within the tube rather than the ovary or endometrium [35]. The recognition that many high-grade serous ovarian cancers originate from the fallopian tube has reshaped our understanding of pelvic serous carcinogenesis, emphasizing the importance of fimbrial sampling and SEE-FIM protocol examinations in surgical pathology [36]. An improved awareness and the early recognition of tubal carcinoma can influence surgical management, staging, and the prognosis, highlighting the need to consider fallopian tube malignancies when postmenopausal adnexal masses display aggressive or complex features.

#### 3.2.3. Secondary (Metastatic) Tumors to the Fallopian Tube

Secondary or metastatic tumors involving the fallopian tube are more common than primary tubal malignancies [37]. This condition represents a crucial component of the differential diagnosis when evaluating adnexal masses with solid characteristics. Metastasis may occur through direct extension, transtubal implantation, hematogenous dissemination, or lymphatic spread [38]. Ovarian serous carcinomas frequently involve the fallopian tube [39]. Endometrial carcinoma may extend into the tube through direct mucosal spread or intraluminal migration [38]. Cervical adenocarcinoma metastasis to the fallopian tube is less common, but may be present in advanced disease [40]. Outside of the gynecologic tract, breast carcinoma, particularly invasive lobular carcinoma, has a predilection for metastasis to Müllerian structures, including the tubes and ovaries [41]. Gastrointestinal cancers such as colorectal adenocarcinoma, gastric signet-ring cell carcinoma (Krukenberg-type metastasis), or appendiceal mucinous tumors may also spread to the fallopian tube and form tumor nodules or mucin-filled lesions that clinically and radiographically resemble ovarian tumors [42]. Metastatic disease tends to present bilaterally more often than primary tumors and may be associated with peritoneal carcinomatosis, ascites, or elevated tumor marker levels [43]. Histopathology plays an essential role in the diagnosis and is particularly valuable for distinguishing metastatic lesions from primary tubal carcinoma using markers such as PAX8, WT-1, the CK7/CK20 profile, and the ER/PR receptor status [44,45], as well as organ-specific markers such as CDX2 for tumors of colorectal origin [46] or mammaglobin/GCDFP-15 for tumors of breast origin [47]. Recognizing metastatic spread is critical for determining the appropriate treatment, as management strategies differ substantially between primary and secondary tumors. Metastatic tumors must be considered in postmenopausal women presenting with a large solid adnexal mass, especially those with elevated tumor markers or evidence of systemic disease. Ultimately, a thorough clinical evaluation combined with a meticulous pathological examination ensures an accurate diagnosis, prevents misclassification, and guides optimal clinical decision-making in patients presenting with fallopian tube tumors (Table 2).

#### 3.2.4. Role of Tumor Markers in Diagnoses and Evaluation

Tumor markers are often used as adjunct tools in the evaluation of adnexal masses to estimate the likelihood of malignancy [48]. However, these methods lack specificity and cannot be used to independently establish a diagnosis. CA-125 is the most commonly used marker of ovarian and tubal pathology [34]. Increased levels are frequently associated with epithelial ovarian cancer [49]. Mild elevation may also be observed in benign conditions such as leiomyomas, endometriosis, or inflammation [50]. The median CA-125 levels typically range from 53 to 413 U/mL in type I ovarian cancer and from 395 to 1340 U/mL in type II ovarian cancer [34]. CEA levels are typically associated with gastrointestinal malignancies and mucinous ovarian tumors [51]. The preoperative serum CEA levels were elevated (>5.0 ng/mL) in 17.5% of patients (10/57), with a median level of 9.6 ng/mL (range, 5.4–111.7 ng/mL) in patients with mucinous ovarian cancer [52]. The SCC antigen and CA19-9 may aid in assessing squamous or mucinous neoplasms, respectively [51,53]. In our case, CA-125 and CEA were only mildly elevated (CA-125 35.4 U/mL, CEA 4.1 ng/mL), while SCC and CA19-9 remained within normal limits—findings that neither confirmed malignancy nor excluded benign disease. Our results highlight that tumor markers primarily serve as supportive indicators and should be interpreted cautiously in the context of clinical presentation and imaging, with a definitive diagnosis ultimately relying on a histopathological examination.

### 3.3. Image Diagnosis

#### 3.3.1. Ultrasound

Transvaginal and transabdominal ultrasound are first-line imaging modalities for evaluating fallopian tube lesions, including paratubal leiomyomas and other adnexal masses [5,54,55]. The American College of Radiology recommends ultrasound as the initial and most appropriate test for suspected adnexal masses, given its superior performance, safety, and cost-effectiveness compared to other modalities [54].

The diagnostic approach involves a systematic assessment of the mass location, size, morphology (solid, cystic, or mixed), and relationship with the ovary and uterus [56]. The use of grayscale and color Doppler imaging to evaluate vascularity helps differentiate benign from malignant lesions. A high color flow and irregular solid components increase the suspicion of malignancy [54]. Standardized risk stratification systems, such as the International Ovarian Tumor Analysis (IOTA) Simple Rules and the Ovarian-Adnexal Reporting and Data System (O-RADS), are used to categorize the risk of malignancy and inform management decisions [33,57]. The IOTA Simple Rules and the O-RADS are validated tools for risk assessment, with the O-RADS providing a five-tiered system for follow-up recommendations [5].

The IOTA Simple Rules system provides a preoperative ultrasound-based method for classifying adnexal tumors, utilizing five sonographic features indicative of benignity (B-features) and five suggestive of malignancy (M-features) [57,58] (Table 3). Tumors are categorized as benign, malignant, or inconclusive when both B and M features are present. B-features include a unilocular cyst, solid components of <7 mm, the presence of acoustic shadows, a smooth multilocular mass of <100 mm, and no detectable blood flow on Doppler imaging (color score 1). M-features include an irregular solid tumor, ascites, ≥4 papillary projections, an irregular multilocular solid mass of ≥100 mm, and a very strong Doppler flow (color score 4). Our case showed a B1 feature (unilocular cyst).

The Ovarian-Adnexal Reporting and Data System (O-RADS) is a standardized ultrasound-based risk stratification framework developed by the American College of Radiology that classifies adnexal masses and guides clinical management [59,60]. It categorizes lesions into six levels based on the morphology, a solid or cystic composition, septations, papillary projections, the Doppler flow, and other sonographic characteristics (Table 4): O-RADS 0 (incomplete evaluation), O-RADS 1 (normal ovary), O-RADS 2 (almost certainly benign; <1% risk), O-RADS 3 (low risk; 1–<10%), O-RADS 4 (intermediate risk; 10–<50%), and O-RADS 5 (high risk of malignancy; ≥50%). The system integrates pattern recognition with a management algorithm, recommending follow-up or surgery based on the risk level, menopausal status, and imaging features. The benign descriptors include simple unilocular cysts and classic hemorrhagic or dermoid cysts, whereas malignant features include irregular solid masses, papillary projections, a strong vascularity, and ascites. The O-RADS improves the diagnostic consistency among clinicians, reduces unnecessary surgery for benign masses, and ensures timely referral for suspicious lesions. It serves as an important tool in the interpretation of adnexal masses, complementing the IOTA Simple Rules and enhancing preoperative decision-making [59]. Our case met the criteria of being solid with a smooth contour of any size and with no flow, which indicates a low risk of ovarian cancer.

Specifically, in paratubal leiomyomas, ultrasound may reveal a well-circumscribed, solid, hypoechoic mass that is adjacent to, but separate from, the ovary and uterus, as demonstrated in our case. In this case, several small calcified spots were observed. A previous study also reported variable calcification patterns in uterine leiomyomas [61]. However, distinguishing paratubal leiomyomas from other solid adnexal masses (e.g., ovarian fibromas and paraovarian cysts) is challenging, and a definitive diagnosis requires a histopathological examination [56,62]. Characteristic sonographic features (e.g., tubular, cystic, or complex masses) and the clinical context aid in diagnosing other fallopian tube lesions, such as a hydrosalpinx, endometriosis, or neoplasms [62]. When ultrasound findings are indeterminate, MRI may be considered for further characterization [5].

#### 3.3.2. CT Scan

Computed tomography (CT) is not typically the primary tool for diagnosing paratubal leiomyomas. In our case, there was a solid, hypovascular tumor with calcified flecks in the left adnexal region and the rightward displacement of the uterus. A previous study demonstrated that intratumoral calcification on CT imaging is a key marker for distinguishing benign from malignant epigastric tumors with a high specificity [63].

CT scans are primarily reserved for specific clinical scenarios, such as acute pelvic pain when the diagnosis is unclear, evaluating complications (e.g., abscess formation), or determining the staging of known malignancies [5,56,64]. CT can identify adnexal masses, hydrosalpinges, pyosalpinges, tubo-ovarian abscesses, and features of isolated fallopian tube torsion. However, its ability to distinguish fallopian tube lesions from ovarian or other adnexal pathologies remains limited. CT findings are often nonspecific, and fallopian tube neoplasms may mimic ovarian carcinomas or other pelvic masses [32,65]. In emergency settings, CT may help identify features such as a U- or C-shaped hydrosalpinx or an extraovarian cyst adjacent to a soft-tissue mass, which can support the diagnosis of isolated fallopian tube torsion [65].

#### 3.3.3. MRI

Compared with ultrasound, which is the first-line modality owing to its accessibility, safety, and cost-effectiveness, MRI is reserved for cases in which ultrasound cannot confidently determine the origin or nature of a lesion. MRI is especially valuable for distinguishing solid from complex cystic masses, identifying hemorrhagic or fatty components, and clarifying the origin of a mass (ovarian vs. tubal vs. other pelvic structures) [66,67,68,69]. For example, MRI can reliably identify hydrosalpinges, hematosalpinges, and tubal neoplasms based on characteristic signal patterns and morphology, and it can differentiate these from ovarian lesions [70,71]. MRI demonstrates a higher specificity and positive predictive value for malignancies than ultrasound or CT in cases with indeterminate findings [5]. While ultrasonography remains the initial test, MRI can reclassify indeterminate lesions, often avoiding unnecessary surgery for benign findings and expediting oncologic referrals for malignant ones [66,72]. In contrast, CT is less sensitive and specific for characterizing adnexal masses and is primarily used for staging known malignancies or assessing complications, not for making an initial diagnosis [5,73].

#### 3.3.4. Positron Emission Tomography–Computed Tomography (PET-CT)

PET-CT is highly sensitive for identifying metabolically active lesions, such as primary fallopian tube carcinoma and metastatic implants, and it can detect sites of disease that may be missed on conventional imaging, as demonstrated in cases where PET-CT identifies lesions that are not visible on MRI or CT [74,75]. However, PET-CT is limited in differentiating between benign and malignant adnexal masses and is not recommended for the initial evaluation or routine diagnosis of benign fallopian tube pathologies [76]. PET/CT is reserved for oncologic indications, such as staging, restaging, and detecting recurrence, and is not suitable for the initial diagnosis or characterization of most fallopian tube lesions [32].

Table 5 provides an overview of the diagnostic imaging approaches used to evaluate fallopian tube and paratubal lesions.

## 4. Conclusions

This case illustrates the diagnostic complexity of a paratubal leiomyoma, an exceedingly rare adnexal tumor that can closely mimic an ovarian malignancy on clinical evaluation and imaging. Despite conducting a thorough assessment using ultrasound, CT, tumor markers, and hysteroscopic sampling, making a preoperative diagnosis of this condition remains uncertain, reflecting the limitations of the current imaging modalities in differentiating uncommon extraovarian smooth muscle tumors from more typical ovarian or tubal pathologies. A definitive diagnosis was achieved through surgical excision and histopathologic confirmation, which demonstrated a hyalinized leiomyoma arising from the paratubal region. This case underscores the importance of maintaining a broad differential diagnosis when evaluating solid adnexal masses, particularly in postmenopausal women, and highlights the crucial role of histopathology in establishing the final diagnosis. Although rare, awareness of paratubal leiomyomas may help avoid overtreatment and guide appropriate surgical planning when adnexal masses present with features suggestive of malignancy.

## Figures and Tables

**Table 1:** Reported cases of fallopian tube/paratubal leiomyomas in the literature.

Author/Year	Patient Age	Presentation	Tumor Location/Size	Imaging Features	Treatment	Pathology/Notes
Yang et al., 2007 [9]	Not specified	Pelvic mass	Fallopian tube; size not stated	Ultrasound showed a solid adnexal mass suggestive of a leiomyoma	Surgical excision	Primary tubal leiomyoma confirmed
Li et al., 2018 [10]	Multiple cases	Varied	Adnexal leiomyomas	Molecular study of MED12 mutation	Surgical specimens reviewed	MED12 mutation is frequently detected in ovarian/adnexal leiomyomas
Misao et al., 2000 [11]	Not specified	Not specified	Fallopian tube leiomyoma	Not reported	Surgical removal	Rare tubal leiomyoma reported
Joshi et al., 2019 [12]	Reproductive age	Ectopic pregnancy with a tubal mass	Fallopian tubal leiomyoma associated with ectopic pregnancy	Not specified	Salpingectomy with pregnancy removal	Coexistence of ectopic gestation and leiomyoma
Sharma et al., 2016 [13]	Not specified	Broad ligament mass	Large cellular leiomyoma with cystic change	Mimicked an ovarian tumor radiologically	Surgical resection	Diagnostic challenge due to cystic degeneration
Sun et al., 2020 [14]	45 years	Abdominal discomfort	Fallopian tube lipoleiomyoma with degeneration	Mass with fatty components	Mass excision	Lipoleiomyoma confirmed histologically
Wu et al., 2024 [8]	49 years	Huge abdominopelvic cystic mass	Fallopian tube origin; very large	Cystic appearance mimicked an ovarian tumor	Surgery performed	Large leiomyoma confirmed
Present case	72 years	Postmenopausal spotting, incidental IUD finding	Left paratubal region; 9.0 × 4.54 × 6.37 cm	Solid mass with calcification on US/CT, mimicking an ovarian malignancy	LTH + BSO; uneventful recovery	Leiomyoma with hyalinization, SMA (+), calretinin (−), inhibin (−)

US, ultrasound; CT, computed tomography; LTH + BSO, laparoscopic total hysterectomy with bilateral salpingo-oophorectomy.

**Table 2:** Summary of differential diagnoses of fallopian tube tumors.

Category	Key Examples	Typical Characteristics	Diagnostic Consideration
Benign tumors	Paratubal/tubal leiomyoma, fibroma, serous/mucinous cystadenoma, papilloma	Usually well-circumscribed solid or cystic mass, often incidental	Often mimics ovarian mass; diagnosis confirmed postoperatively
Primary malignant tumors	HGSC, LGSC, endometrioid, clear cell, mucinous carcinoma, leiomyosarcoma, MMMT	Imaging similar to ovarian cancer; CA-125 may be elevated	Requires histological proof of tubal origin; SEE-FIM recommended
Secondary (metastatic) tumors	From the ovary, endometrium, breast, colorectal/appendix	More common than primary; often bilateral or associated with peritoneal disease	IHC critical to identify primary site; alters management strategy

HGSC, high-grade serous carcinoma; LGSC = low-grade serous carcinoma; MMMT = malignant mixed Müllerian tumor; SEE-FIM = sectioning and extensively examining the FIMbria protocol; IHC, immunohistochemistry.

**Table 3:** International Ovarian Tumor Analysis (IOTA) Simple Rules for adnexal mass classification.

Category	Feature Type	Ultrasound Criteria
B-features (benign indicators)	B1	Unilocular cyst
	B2	Presence of solid components of <7 mm
	B3	Presence of acoustic shadows
	B4	Smooth multilocular tumor <100 mm in largest diameter
	B5	No detectable blood flow on color Doppler (color score of 1)
M-features (malignant indicators)	M1	Irregular solid tumor
	M2	Presence of ascites
	M3	≥4 papillary projections
	M4	Irregular multilocular solid tumor of ≥100 mm
	M5	Very strong blood flow on Doppler (color score of 4)

**Table 4:** Ovarian-Adnexal Reporting and Data System (O-RAD) categories.

O-RADS	Features	Risk
1	Follicles <3 cm, corpus luteum (thickened wall <3 cm)	Normal ovary (0% likelihood of malignancy)
2	Simple cyst (>3–<10 cm in premenopausal women, <10 cm in postmenopausal women); not simple, unilocular cyst with smooth inner margin <10 cm; classic benign lesions (hemorrhagic cyst, dermoid cyst, endometrioma, paraovarian cyst, peritoneal inclusion cyst, hydrosalpinx)	Almost certainly benign (<1% likelihood of malignancy)
3	Unilocular cyst ≥10 cm; typical benign cyst ≥10 cm; unilocular cyst with irregular inner wall (<3 mm); multilocular cyst with smooth inner wall (<10 cm) and low color flow; solid with smooth contour, of any size and with no flow	Low risk (1–<10% likelihood of malignancy)
4	Multilocular cyst with smooth inner wall, ≥10 cm, no-to-moderate flow; multilocular cyst with smooth inner wall of any size and a very strong flow; multilocular cyst with irregular inner wall or septation of any size and any flow; unilocular cyst with 1–3 papillary projections of any size and any flow; multilocular cyst with solid component of any size and no to mild flow; solid (≥80%) with smooth contour of any size and mild-to-moderate flow	Intermediate risk (10–<50% likelihood of malignancy)
5	Unilocular cyst with ≥4 papillary projections of any size and any flow; multilocular cyst with solid component of any size and moderate-to-strong flow; solid (≥80%) with smooth contour of any size and a very strong flow; solid (≥80%) with irregular contour of any size and any flow; ascites and peritoneal nodules	High risk (≥50% likelihood of malignancy)

**Table 5:** Summary of imaging modalities for fallopian tube and paratubal lesions.

Imaging Modality	Advantages/Role	Key Diagnostic Features	Limitations/Notes
Ultrasound (TVS/TAS)	First-line for adnexal evaluation; recommended by ACR; cost-effective and widely available	Assesses mass size, morphology, and vascularity (Doppler); IOTA rules/O-RADS applicable. Paratubal leiomyoma appears as a solid, well-circumscribed, hypoechoic mass separate from the ovary/uterus	Difficult to distinguish between ovarian fibromas and paraovarian cysts; operator-dependent; definitive diagnosis requires pathology
CT scan	Useful for acute settings, complication evaluations, and malignancy staging	Detects adnexal masses, hydrosalpinges, pyosalpinges, TOAs, and torsion indicators (U-/C-shaped tube)	Poor discrimination between tubal vs. ovarian tumors; nonspecific for benign vs. malignant; not preferred for initial diagnosis
MRI	Best for characterization of indeterminate masses after US; high specificity for malignancy	Differentiates solid vs. cystic components; identifies hemorrhage/fat; clarifies origin (tubal vs. ovarian)	More expensive and less accessible; reserved for uncertain cases; not first-line, despite superior characterization
PET-CT	Highly sensitive for metabolically active malignancy, metastasis, and recurrence evaluations	Detects sites missed by CT/MRI; useful for oncologic staging	Not suitable for initial diagnosis of benign lesions; limited ability to differentiate benign from malignant adnexal masses

TVS/TAS, transvaginal sonography/transabdominal sonography; US, ultrasound; CT, computed tomography; MRI, magnetic resonance imaging; PET-CT, positron emission tomography–computed tomography; ACR, American College of Radiology; IOTA, International Ovarian Tumor Analysis; O-RADS, Ovarian-Adnexal Reporting and Data System; TOA, tubo-ovarian abscess.

## Data Availability

No new data were created or analyzed in this study. Data sharing does not apply to this study.

## Abbreviations

The following abbreviations are used in this manuscript:

BSO	Bilateral salpingo-oophorectomy
CA-125	Cancer antigen 125
CEA	Carcinoembryonic antigen
CT	Computed tomography
HGSC	High-grade serous carcinoma
HPV	Human papillomavirus
IHC	Immunohistochemistry
IOTA	International Ovarian Tumor Analysis
IUD	Intrauterine device
LTH	Laparoscopic total hysterectomy
MRI	Magnetic resonance imaging
O-RADS	Ovarian-Adnexal Reporting and Data System
PET-CT	Positron emission tomography–computed tomography
SCC antigen	Squamous cell carcinoma antigen
SMA	Smooth muscle actin
